# Development of a Dual-Arm Robotic Cell for Automated Manufacturing of Variable-Geometry Thermoplastic Composite Ducts

**DOI:** 10.3390/s26196152

**Published:** 2026-09-28

**Authors:** Ali Chokre, Ahmed Joubair, Simon Joncas, Jean-Philippe Roberge

**Affiliations:** Control and Robotics Laboratory, École de Technologie SupéRieure, Montreal, QC H3C 1K3, Canada; ali.chokre.1@ens.etsmtl.ca (A.C.); cc-ahmed.joubair@etsmtl.ca (A.J.); simon.joncas@etsmtl.ca (S.J.)

**Keywords:** dual-arm robotics, thermoplastic composites, laser welding, automated trajectory generation, aerospace manufacturing

## Abstract

This research focuses on the development of a robotic cell test bench for manufacturing variable-geometry tubular parts with minimal manual intervention. The system integrates dual-arm manipulation with cutting and laser-welding stations and was evaluated in collaboration with an industrial partner with strict requirements on joint strength and sealing performance. The experimental investigation characterized the influence of key welding process parameters, particularly the robot end-effector travel speed (mm/s) and laser power (W), on the mechanical performance of the welded joints. These parameters were subsequently used to manufacture representative three-dimensional duct geometries. In addition, preliminary sealing evaluations were used to assess the ability of the welded components to withstand internal pressures. These results highlight the influence of mechanical components, geometries, and material behavior, as well as the need for advanced digital tools. A preliminary automated path-generation methodology is introduced to support adaptable robot trajectories. Overall, the results support the feasibility of the integrated robotic manufacturing concept while identifying the additional developments required before fully automated production can be achieved.

## 1. Introduction

In recent years, research and industry have increasingly focused on the forming and processing of thermoplastic composite parts, driven in particular by the growing demand for lightweight, high-performance materials in sectors such as aerospace and automotive [1]. Compared with thermoset composites, thermoplastic composites offer several advantages, including shorter forming and consolidation times, chemical stability, recyclability, and weldability [2,3]. Despite these advantages, their industrial adoption, particularly in the aerospace sector, remains constrained by challenges related to manufacturing automation.

The manufacturing of thermoplastic composite parts in the aerospace industry remains largely dependent on manual operations. This is primarily due to the complexity of the manufacturing processes, the diversity of component geometries, and the relatively low production volumes characteristic of the sector [4,5]. While conventional industrial robotic systems are well suited to high-volume and highly repetitive operations, they often lack the flexibility required for small production batches and frequent changes in part geometry [6]. Introducing a new component may require substantial engineering effort, including the reprogramming of tool paths, adaptation of end-effectors, cell reconfiguration, process-speed calibration, and generation of new simulations and robot instructions. In addition, the design and fabrication of specialized tooling for individual part geometries can further reduce the economic viability of robotic automation. These constraints become increasingly significant as robots are expected to accommodate a wider range of parts, tasks, and trajectories.

This study addresses two key aspects toward advancing the automation of thermoplastic composite part manufacturing. First, it investigates the influence of process parameters, including operating speed, welding power, and material configuration, on the quality of components produced using robotic manufacturing techniques. Sample parts are manufactured and mechanically tested to assess and validate their performance. Second, this study presents the development of a numerical tool for automated robot trajectory generation, intended as a step toward a more comprehensive digital workflow for robotic composite manufacturing.

The underlying dual-arm robotic-cell architecture and part of the associated tooling were developed in previous work [7,8]. Building on this foundation, the present work makes three main contributions:

(i) The application-specific integration of the two industrial robots with the cutting and laser-welding stations into a unified manufacturing workflow for variable-geometry thermoplastic composite ducts;
(ii) The development of an initial CAD-based pipeline for generating discrete robot poses from STEP geometry and transferring them to an offline programming environment;
(iii) The experimental characterization and preliminary feasibility validation of the integrated manufacturing workflow through welding-parameter studies, mechanical testing, the fabrication of straight and curved duct specimens, and sealing tests.

Accordingly, the novelty of this work lies in the combined application-specific implementation, numerical workflow and experimental assessment. The trajectory generation component remains at the prototype stage and requires further integration and experimental validation.

The remainder of this paper is organized as follows. In Section 2, the literature review examines thermoplastic composite manufacturing and automated robot programming. The objectives, presented in Section 3, then define the scope of this study. The robotic cell development, in Section 4, describes the experimental equipment, trajectory-generation pipeline, and validation of the cutting and welding processes. Finally, the conclusions presented in Section 5 summarize the main findings and directions for future work.

## 2. Literature Review

### 2.1. Welding of Thermoplastic Composite Materials

Thermoplastic composites are increasingly used in aerospace applications because of their high specific strength, low weight, and potential for automated manufacturing. The robotic cell developed in this work targets key manufacturing operations, including thermoforming, cutting, and welding. Although these processes are well established, their operating parameters must be adapted and optimized to meet the requirements of the targeted industrial application. Important thermoforming parameters include heating temperature, applied pressure, and consolidation time, whereas welding performance is primarily influenced by parameters such as applied power, pressure, and travel speed. Understanding the effects and interactions of these parameters requires consideration of both the manufacturing process and the underlying material behavior.

The use of composite materials in aerospace applications requires specialized manufacturing and qualification procedures [9]. Polymer matrix composites (PMCs) consist primarily of a reinforcing phase, which provides strength and stiffness, and a polymer matrix, which binds the reinforcement and transfers loads between reinforcing elements. Thermosetting matrices undergo irreversible curing, whereas thermoplastic matrices can be softened upon heating and subsequently reshaped, providing greater flexibility for manufacturing and assembly. Figure 1 illustrates a composite plate and its main constituent elements.

In the targeted application, the manufacturing of thermoplastic composite fluid-transfer ducts remains largely dependent on manual operations and lacks a standardized automated production process. Operations such as cutting, welding, thermoforming, and hot stamping must therefore be investigated and adapted for robotic manufacturing. Previous developments of the robotic cell and associated tooling have been reported in [7] and by Sauvain [8]. The present work builds upon these developments by further investigating process parameters and automated manufacturing strategies. Because aerospace ducts must satisfy stringent functional requirements, their qualification typically includes mechanical-strength and sealing tests.

The work of Côté [7] focused on the development of the robotic cell and its tooling, including the creation of 3D CAD models and the integration of the cell’s modules within a dedicated offline programming software, along with the introduction of the fundamental manufacturing methods. In parallel, the work of Sauvain [8] addressed the cell’s actual configuration and software architecture, including controller-level communication between the two robots (i.e., a master–follower architecture), followed by the programming of the robot tool paths and associated logic handling (I/Os, safety checks, and robot post-processor adaptation). The respective contributions of [7,8] can thus be seen as laying, respectively, the hardware and software foundations of the dual-arm robotic cell. As outlined in the Introduction, the present work builds on these foundations and focuses on experimental testing and process improvement, the evaluation of selected process parameters, and preliminary numerical methods for automatic robot tool-path generation, aimed at enabling rapid adaptation and flexibility in the manufacturing of variable-geometry composite ducts.

Among the available joining methods, laser welding represents a promising solution for automating the assembly of thermoplastic composite structures, particularly for joining and sealing half-shell components. Successful laser welding requires precise control of process parameters, notably welding speed (mm/s) and laser power (W), to achieve joints that satisfy mechanical requirements. Suitable operating conditions may be identified through experiments or numerical modeling; however, experimental characterization remains necessary to validate the resulting joint quality. Because welding behavior depends strongly on material properties, joint configuration, heat transfer, and process conditions, no universal set of parameters can guarantee defect-free joints across all thermoplastic composite applications.

Several joining strategies can be considered for thermoplastic composites, as illustrated in Figure 2. Mechanical fastening is widely used in aerospace because it provides reliable structural connections; however, bolts, screws, and rivets add weight, require additional manufacturing operations, and may introduce local stress concentrations. Adhesive bonding can provide a favorable strength-to-weight ratio while simplifying joint geometry [3]. Nevertheless, adhesive processes require careful control of factors such as curing or drying conditions, humidity, temperature, and surface preparation, which may complicate their integration into a fully automated manufacturing process. Welding is therefore particularly attractive for robotic processing of thermoplastic composites because it can rapidly create joints without requiring additional fastening material. Most thermoplastic welding techniques rely on locally heating the polymer matrix above its melting temperature while applying pressure to promote intimate contact and consolidation at the interface [2].

Mechanical characterization of welded samples is essential for validating the manufacturing process. The appropriate evaluation method depends on the geometry, function, and expected loading conditions of the component. Bonded and welded interfaces can generally be subjected to three fundamental fracture-loading modes: opening (Mode I), in-plane shear (Mode II), and out-of-plane shear (Mode III) [12]. For the type of lap joint considered in this work, tensile loading can be used to characterize the mechanical resistance of the welded interface and quantify its apparent shear strength. Tensile tests are used to quantify the shear strength of welded joints, typically expressed in terms of stress σ and strain ε.

### 2.2. Generation of Robot Tool Paths

The development of a flexible robotic cell for cutting and laser-welding-reinforced thermoplastic components requires programming methods capable of reducing the engineering effort associated with conventional manual robot programming, particularly for low-volume production involving frequent variations in part geometry. CAD-to-Path (C2P) approaches address this challenge by extracting geometric information from CAD models and using it to generate tool trajectories and robot instructions through numerical processing and simulation. Such approaches constitute an important component of Automatic Offline Programming (AOLP).

In conventional Offline Programming (OLP), robot trajectories are generally defined within a simulation environment by manually selecting geometric features and specifying process-related waypoints. Wu et al. [13], for example, demonstrated such an approach for robotic welding applications. More recent developments combine OLP environments with Application Programming Interfaces (APIs), allowing portions of the programming workflow to be automated and facilitating the implementation of trajectory-generation and optimization algorithms.

Several methods have been proposed for automated trajectory generation in welding and surface-processing applications. For example, ref. [14] introduced a waypoint-generation strategy based on two- and three-dimensional interpolation of meshed CAD geometries. Although this approach contributes toward automated CAM development, its implementation remains dependent on commercial CAD software (SolidWorks 2021) and MATLAB 2022a. Similarly, IRoSim [15], developed using the SolidWorks API and shown in Figure 3, integrates mechanical design and robotic simulation within a unified CAD environment. While this framework facilitates robot programming, it remains dependent on proprietary software and requires trajectory information to be redefined when the geometry of the manufactured component changes. Other CAD-based approaches, such as [16], reduce the required user input by defining a limited set of waypoints and registering the CAD model with the physical robotic setup. However, such methods are not specifically designed to automatically generate the complex curved trajectories encountered in thermoplastic composite duct manufacturing.

These limitations highlight the need for lightweight, open-source numerical tools capable of generating complete robotic trajectories from CAD geometry and a limited number of high-level process parameters. Reducing the amount of application-specific manual programming constitutes an important step toward more automated offline programming workflows.

An additional consideration arises from the configuration of the robotic cell investigated in this work. Most conventional trajectory-planning approaches assume that the workpiece remains fixed while a robot-mounted tool follows the desired processing path. In the proposed setup, however, the thermoplastic component is mounted on the robot end-effector and manipulated relative to a stationary processing tool. Consequently, the desired manufacturing trajectory must be expressed in terms of the motion of the workpiece rather than that of the tool. This configuration introduces additional considerations related to coordinate transformations, robot kinematics, and velocity control, particularly because the relative velocity between the workpiece and the processing tool must remain consistent with the prescribed process parameters.

Standardized CAD representations provide a useful basis for addressing these challenges. STEP files, for example, can represent complex geometries, including Non-Uniform Rational B-Spline (NURBS) surfaces, within the ISO 10303 framework [17,18]. Geometry-processing libraries such as Open Cascade Technology (OCCT) can then be used to access and manipulate this CAD information programmatically. Bedaka and Lin [19] demonstrated the use of such tools for automated processing of CAD geometry without requiring direct manipulation of the underlying STEP file representation. This type of approach is particularly attractive for developing software-independent trajectory-generation tools for robotic thermoplastic composite manufacturing.

More broadly, AOLP research increasingly focuses on extracting geometric and semantic information directly from CAD models to minimize user intervention and automatically generate executable robot trajectories. Representative applications include automated spray-painting trajectories over complex surfaces [20], adaptive tool-path generation for machining complex geometries [21], and semi-automated inspection-path planning for aerospace non-destructive testing [22]. These studies demonstrate the potential of CAD-driven robot programming across a variety of manufacturing processes.

Recent work shows that progress toward a complete AOLP workflow can be analyzed along three complementary dimensions. The first is trajectory authoring and data interoperability. Babcinschi et al. [23], for example, combined positions extracted from CAD/CAM with tool orientations and velocities captured from a single expert demonstration and used the AutomationML-based PathML format to transfer process and robot data across the programming workflow. Their experimental welding and adhesive-deposition trials quantified position and orientation errors, but the method still relies on an expert demonstration and virtual-to-real calibration. The second dimension is quantitative performance assessment and system-level optimization. Kawabe et al. [24] jointly optimized robot motion and cell layout using RRT*, NSGA-II, and a surrogate model, with cell area, robot operation time, computation time, and surrogate accuracy as explicit performance criteria. The third dimension concerns robust optimization under uncertainty. Although not specific to robot programming, the sequential optimization and time-dependent reliability assessment method proposed by Zhang et al. [25] illustrates how deterministic optimization and reliability analysis can be decoupled to reduce the computational cost of design optimization under time-dependent uncertainty.

Taken together, the literature indicates a progression from application-specific path construction, through interoperable program generation and simulation-based validation, toward the joint optimization of robot motion and manufacturing-cell performance. However, these capabilities are generally distributed across separate frameworks. CAD-driven methods reduce manual waypoint definition but remain dependent on application-specific geometric processing; demonstration-based methods capture process expertise but require operator input and calibration; and system-level optimization methods require explicit objective functions, constraints, and performance data. Table 1 provides a structured comparison of the reviewed approaches in terms of their programming input, performance, or optimization focus and principal limitation relative to the variable-geometry duct application considered here.

Despite these advances, no generalized or standardized AOLP methodology has emerged across robotic manufacturing applications. Although the literature exhibits broad similarities in the principal stages of an automated programming workflow, the methods used to extract geometry, identify relevant features, generate process trajectories, and convert these trajectories into executable robot commands remain highly application dependent. This motivates the development of the trajectory-generation approach presented in this work, which targets the specific requirements of robotic cutting and laser welding of thermoplastic composite components.

The following sections present the research objectives, the development of the proposed trajectory-generation tool, the robotic cell and experimental setup, and the resulting experimental validation.

## 3. Objectives

The primary objective of this research is to demonstrate the feasibility of automating the manufacture of thermoplastic composite fluid-transfer ducts while accommodating the substantial geometric variability encountered in aerospace applications. To achieve this objective, the robotic manufacturing cell must be capable of adapting to different duct geometries while satisfying the process and mechanical requirements of the targeted application. Figure 4 illustrates a typical aircraft ducting system and highlights the geometric diversity of the components required for cabin air distribution.

This general objective is addressed through two complementary aspects. First, a numerical trajectory-generation approach is developed to reduce the programming effort required when adapting the robotic cell to different part geometries. The objective is to automatically derive suitable robot trajectories from the geometry of the component while accounting for the process parameters required for cutting and welding operations. Second, the manufacturing process is experimentally characterized to determine the influence of key operating parameters, particularly welding power and travel speed, on the mechanical performance of the resulting joints.

The robotic cell, its associated tooling, and the proposed programming approach are then evaluated through the manufacturing and mechanical characterization of representative thermoplastic composite samples and components. In particular, the mechanical resistance of welded joints is assessed to compare the tested processing conditions. Together, these developments aim to demonstrate the potential of the proposed robotic cell as a flexible manufacturing platform for thermoplastic composite ducts with varying geometries.

## 4. Robotic Cell Development

This section presents the development of the robotic manufacturing cell and the two complementary research activities investigated in this work. The first concerns the initial development of a numerical pipeline for automated trajectory generation, intended to reduce the programming effort required when adapting the cell to different duct geometries. The second focuses on the development and experimental validation of the cutting and welding processes through the fabrication and mechanical characterization of representative thermoplastic composite components.

Figure 6 shows an elevation view of the 3D model of the robotic cell, highlighting the main configurations associated with the operating stations. Each configuration corresponds to a specific set of joint values that brings a robot to a given reference position. Each configuration is identified by the number of the corresponding operating station. For example, ST12 is the position of Robot 1 (R1) at the cutting station, while ST30 is the position of either robot at the laser-welding station. ST10 and ST20 are the initial positions required by R1 and Robot 2 (R2), respectively, for loading the raw half-shells.

Figure 5 presents the manufacturing sequence used to produce a composite duct sample with an auxiliary connector. It should be read together with Figure 6 which shows the layout of the operating stations and the corresponding robot configurations. Each transition in the sequence is labeled according to the configuration that the robot must reach to perform the next operation. The two half-shells are processed in parallel with one held by each robot manipulator. R1 and R2 start from their initial positions and move to ST10 and ST20, respectively, for manual loading of the raw composite half-shells. They then move to ST12 and ST22 for ultrasonic trimming at their dedicated cutting stations. The two sequences subsequently differ. The half-shell handled by R1 undergoes auxiliary hole cutting and spot welding of the connector. The half-shell handled by R2 proceeds directly to the assembly configuration and waits there. R1 and R2 finally reach ST30 where the two half-shells are brought together and joined by laser transmission welding (LTW). The illustrated workflow terminates at ST30 with the completed duct still supported by the two robots after welding.

### 4.1. Research Equipment

The robotic cell consists of two industrial six-degree-of-freedom (6-DOF) manipulators and two composite cutting stations. Each robot has a dedicated cutting station. A laser-welding station is positioned between the two manipulators. Robot trajectories and control programs are currently developed using RoboDK 5.6.9 and its API under Python 3.10, enabling offline simulation, trajectory validation, and generation of executable robot programs.

For mechanical characterization, two primary test benches were employed. Figure 7 illustrates the experimental setup used for tensile testing of the welded specimens. The joint-breaking load (N) was evaluated for both 2D and 3D duct samples. Parameter combinations yielding the highest joint-breaking loads were selected for further investigation. Shear tests were performed on 2D duct specimens, with four samples tested for each welding parameter set using an INSTRON 5980 Series universal testing machine (Instron, Norwood, MA, USA). Tensile loading was applied in accordance with the ASTM D3039 standard [27] for polymer matrix composite materials, using a crosshead displacement rate of 0.08 inches per minute. This standard defines the complete testing procedure, including specimen geometry, loading rate, surface preparation, gauge length positioning, and pre-load conditions. The standard is particularly appropriate for thin composite laminates reinforced with fibers having a high modulus.

In the demonstrated workflow, each robot carries one half-shell to its dedicated cutting station. The two robot-held components are then moved directly to the central laser-welding station, without a conveyor or an intermediate transfer between grippers. During welding, the Master–Follower motion maintains their relative pose while the joint is moved with respect to the stationary laser tool. The present study does not evaluate station-to-station transfer times or production throughput.

The leak-test setup shown in Figure 8 enables the ducts to be pressurized under controlled conditions and provides a means of assessing their sealing performance and mechanical integrity. Both qualitative leak detection and pressure-decay measurements are used as preliminary indicators of the ability of the manufactured ducts to satisfy the functional requirements of the targeted application.

### 4.2. Initial Trajectory-Generation Pipeline

Automated robot trajectory generation is an important component of a flexible manufacturing cell intended for low-volume production involving substantial part variability. In this work, a numerical pipeline is developed to generate manufacturing trajectories directly from the CAD geometry of fluid-transfer ducts, thereby reducing the amount of manual robot programming required for each new component. The approach processes a three-dimensional CAD model and generates a discrete sequence of robot poses describing both the position and local orientation (Yaw, Pitch, and Roll) required along the manufacturing trajectory. The resulting trajectory is subsequently transferred to RoboDK for simulation, validation, and robot-code generation. The proposed trajectory-generation pipeline consists of the following main steps:

1. Importing the three-dimensional model from a STEP file;
2. Extracting the relevant geometric primitives from the model;
3. Computing an Oriented Bounding Box (OBB) based on the part geometry;
4. Generating the welding plane and its associated normal vector;
5. Redefining the global reference frame according to the part geometry;
6. Discretizing the central trajectory of the part;
7. Defining the local orientation at each trajectory point;
8. Preparing the geometric pose sequence for subsequent robot-specific transformation within the offline programming environment; and
9. Exporting the resulting trajectory to the offline programming environment for validation, simulation, and robot-code post-processing.

Figure 9 highlights the three key steps of the path generation algorithm. It is important to note that the main components of a discrete path can be defined using the Frenet frame formulation. Each discrete point along the trajectory is represented by a 3D position coordinate (*x*, *y* and *z*) and an orientation defined by three orthogonal unit vectors (tangent, normal, and binormal), forming a local coordinate system at each point along the robot path. This discretization can be expressed using the following 4 × 4 transformation matrix $F_{k}$:

(1) $$F_{k}=\left[\begin{array}{cc} R_{k} & P_{k}\\ 0 & 1 \end{array}\right]=\left[\begin{array}{cccc} T_{k} & P_{k}^{⊥} & B_{k} & P_{k}\\ 0 & 0 & 0 & 1 \end{array}\right].$$

For a differentiable central curve C(u), the position and local Frenet basis at the sampled parameter value $u_{k}$ are defined as

(2) $$\begin{array}{cc} P_{k} & =C(u_{k}),\\ T_{k} & =\frac{C^{\prime }\left(u_{k}\right)}{∥C^{\prime }\left(u_{k}\right)∥},\\ P_{k}^{⊥} & =\frac{T^{\prime }\left(u_{k}\right)}{∥T^{\prime }\left(u_{k}\right)∥},\\ B_{k} & =T_{k}\times P_{k}^{⊥}. \end{array}$$

$P_{k}\in R^{3}$ represents the position of the *k*th trajectory point, while $T_{k}$, $P_{k}^{⊥}$ and $B_{k}$ are three-dimensional unit vectors defining the tangent, normal, and binormal directions, respectively. These vectors form the rotation matrix $R_{k}$ and therefore define the local orientation associated with each trajectory point. The resulting sequence of homogeneous transformations provides the pose information required for subsequent trajectory adaptation, simulation, and robot-program generation.

In the current implementation, the STEP geometry is processed using OCCT, and the oriented bounding box is used to establish a part-aligned reference frame. The welding plane and its normal provide the reference directions used to identify the central path. This path is represented as a parametric curve C(u) and sampled at successive parameter values $u_{k}$ to obtain the discrete positions $P_{k}=C\left(u_{k}\right)$. The local curve direction provides the tangent vector, while the associated normal and binormal vectors define the orientation used in $F_{k}$. The influence of the sampling interval was not investigated or optimized in this study; consequently, the current work does not establish a geometry-independent discretization step.

The current output is a geometric pose sequence rather than a fully time-parameterized, coordinated dual-robot motion. The local frames are subsequently processed by interpolating the orientations between successive sampled points to preserve angular continuity along the trajectory. This treatment limits abrupt changes in frame orientation near regions of low curvature or changing curvature direction. It uses direct interpolation rather than a dedicated alternative moving frame formulation. Velocity-profile planning and automated transformation into the calibrated base frames of both robots are not included in the present pipeline. Collision, reachability, and singularity checks, as well as process-speed assignment and robot-specific coordinate transformations, are performed after export within the RoboDK environment and the existing Master–Follower programming workflow. These limitations are consistent with the preliminary scope of the trajectory-generation tool and define the main requirements for its subsequent integration with the physical cell.

The evaluation criteria are defined separately for the manufacturing process and the preliminary trajectory-generation pipeline. Manufacturing performance is assessed using the mean joint-breaking load (kN), obtained from four tensile specimens for each welding-parameter combination; the completion of automatic manufacturing runs without manual intervention or process-related downtime; and preliminary sealing performance, based on the maximum applied internal pressure and the measured pressure decay.

The automated trajectory-generation pipeline was not used to generate the paths employed in the manufacturing trials and has not yet been fully integrated with the physical cell. Consequently, programming-time reduction, physical trajectory accuracy, and cycle time were not measured in the present study. At this preliminary stage, the pipeline is evaluated qualitatively by verifying that it generates a complete sequence of discrete poses with coherent orientations that can be transferred to RoboDK for simulation and post-processing. Quantitative benchmarking of these metrics is therefore identified as part of the next development phase rather than claimed as an outcome of the present work.

Figure 10 summarizes the technical roadmap of this study. The work builds on the dual-arm robotic cell developed in Refs. [7,8] and follows two parallel development pillars. The first addresses physical process development and experimental validation using conventional OLP. The second addresses development of the preliminary AOLP pipeline. These pillars are evaluated separately in the present study while their full integration into the physical manufacturing workflow is reserved for future work.

### 4.3. Development and Validation of the Cutting and Welding Processes

The second component of this work concerns the fabrication and experimental validation of parts produced using the robotic cell. Four part configurations were considered, encompassing both straight (2D) and curved (3D) reinforced thermoplastic ducts, as well as foam-based duct components. Figure 11 presents the three-dimensional model of a representative reinforced thermoplastic duct. Each duct consists primarily of two half-shells, denoted R1 and R2, which are handled by robots R1 and R2, respectively.

Dual-arm manipulation is used to cooperatively position and assemble the two half-shells within the multi-station robotic cell. Accurate control of the relative pose between the two manipulators is particularly important during laser welding and during the associated approach, positioning, and repositioning motions. As illustrated in Figure 12, a Master–Follower control strategy is used, with robot R1 acting as the guiding robot and robot R2 following the coordinated motion. Each manipulator holds one half-shell while the two components are positioned to form the complete duct and expose the interfaces required for welding.

Within this conventional OLP workflow, the motion of robot R1 defines the reference trajectory, while robot R2 maintains the prescribed relative pose between the two half-shells through the existing Master–Follower controller architecture described in Ref. [8]. Inverse kinematics are solved using RoboDK and the native robot controllers. Before program transfer, the simulated trajectories are checked for reachability, joint limits, singularities, and collisions with the modeled cell components. The present work does not introduce a new inverse-kinematics solver or a real-time collision-avoidance module; collision-risk management relies on offline validation and the controller-level safety logic of the existing cell.

Although the broader cell architecture was conceived to accommodate additional operations, including coating, the experimental workflow reported here focuses on cutting and laser welding. Using nominal welding parameters of 100 W and 12 mm/s, testing was extended to 3D ducts. After minor adjustments to robot speed and tooling clearance, successful fabrication of 3D geometries was achieved. Under the final setup, four production runs—one 3D Polyetherimide (PEI) duct with SPUD and three reinforced thermoplastic ducts—were completed in fully automatic mode without human intervention or process-related downtime, corresponding to a completion rate of 4/4 for this limited demonstration set. This result is reported as a feasibility indicator rather than as an estimate of industrial production reliability.

In this context, “without human intervention” refers specifically to execution of the programmed manufacturing sequence after the parts, tooling, process parameters, and robot programs have been prepared and validated. Initial part loading and fixturing, cell calibration, offline program preparation, process-parameter selection, and post-process inspection remain operator-assisted activities. The automation claim therefore concerns the execution of the coordinated cutting and welding operations rather than fully autonomous end-to-end production.

The robotic cell uses laser transmission welding (LTW) with a near-infrared (NIR) laser to locally heat the joint interface. As illustrated in Figure 13, the laser radiation passes through the semi-transparent layer and is absorbed by the underlying absorbing (black) layer. The resulting localized heating raises the temperature at the interface and enables fusion of the thermoplastic material under applied contact pressure.

The influence of welding parameters was initially investigated using 2D duct specimens. Laser power and robot travel speed were varied to determine operating conditions providing adequate mechanical resistance of the welded joint. Four specimens were tested for each parameter combination using an INSTRON 5980 Series universal testing machine at a crosshead displacement rate of 0.08 in/min. Tensile loading was applied to the welded specimens, and the breaking load of the joint was used as the primary metric for comparing welding conditions.

The results are typically expressed in terms of stress σ and strain ε. Equations (3) and (4) provide these values. Stress (σ) is calculated by dividing the applied force (*F*) by the original cross-sectional area perpendicular to the load direction ($A_{0}$), while strain (ε) is the ratio of the elongation (*l* − $l_{0}$) to the initial length ($l_{0}$).

(3) $$\sigma =\frac{F}{A_{0}}$$

(4) $$\varepsilon =\frac{l-l_{0}}{l_{0}}$$

Figure 14 summarizes the experimental workflow. First, the dual-arm robotic cell manufactures the duct specimens using selected combinations of robot travel speed and NIR laser power. The resulting parts are then visually inspected to identify apparent welding defects. Finally, the ducts are sectioned into specimens for mechanical testing, and the resulting breaking loads are recorded for analysis.

Figure 15 shows the mean joint-breaking load (kN) for the tested parameter combinations. Each group of bars corresponds to a different laser power. Among the tested conditions, a laser power of 100 W combined with a travel speed of 12 mm/s produced the highest average breaking load, approximately 1.5 kN. These parameters were therefore selected as the nominal welding conditions for subsequent experiments.

No valid result was available for the 120 W and 6 mm/s combination; its absence from Figure 15 should therefore not be interpreted as a zero breaking load. The archived dataset retains only the mean of the four specimens for each condition and not the individual breaking load values. Standard deviations and confidence intervals therefore cannot be calculated retrospectively. The comparison is consequently treated strictly as descriptive parameter screening. The 100 W and 12 mm/s condition is identified only as the highest mean among the tested conditions rather than as a statistically validated optimum.

Increasing laser power beyond this point likely leads to excessive heating and thermal degradation of the polymer matrix, resulting in reduced weld shear strength. These results indicate that thermal degradation dominates at higher power levels, emphasizing the importance of maintaining a delicate balance between sufficient fusion and overheating. This highlights the narrow processing window of thermoplastic laser welding and the need for precise control of both power and travel speed. In the air leak-test procedure, the duct is installed on the test bench and gradually pressurized in 2.5 psi steps, starting at 2.5 psi and increasing up to a maximum of 15 psi. At each step, the duct is inspected by spraying a soap-water solution on its surface to detect leaks, indicated by the formation of air bubbles as shown in Figure 16. These pressure levels are successive stages of a single loading sequence applied to one representative 2D duct; they are therefore not treated as independent experiments or as evidence of repeatability.

The tested 2D duct withstood the maximum applied pressure of 15 psi without visible structural degradation, but the soap-bubble inspection revealed localized leakage. This stepwise pressurization test did not include a standardized holding period at 15 psi and did not provide a calibrated volumetric leakage rate. It therefore indicates pressure resistance but does not demonstrate compliance with a sealing requirement.

A separate pressure-decay test was conducted using the same test bench. After pressurization, the air supply was shut off, and the internal pressure was recorded over time. As shown in Figure 17, the measured pressure decreased from approximately 2.8 psi at t=0 to approximately 0 psi after 330 s. The corresponding average pressure-decay rate over the full interval was approximately 2.8/330=0.0085 psi/s. Because the decay is nonlinear, this value is reported only as an overall indicator rather than as a constant leakage rate.

The available pressure-decay measurement cannot be converted into the normalized volumetric leakage rate commonly used for duct qualification because the test did not provide calibrated continuous flow data or normalization by the effective duct area. Moreover, the acceptance threshold supplied by the industrial partner is proprietary and application specific. A direct comparison with that threshold therefore cannot be disclosed, and the present results are treated as a preliminary sealing assessment rather than an industrial qualification test.

Figure 18 shows the final outcome of a reinforced thermoplastic 3D fluid transfer duct produced using the selected welding parameters. The foam cutting station also presented challenges due to material behavior during cutting. The force generated by the interaction between the oscillating blade and the foam caused deformation and tearing in the cutting area, rather than producing a clean cut. Figure 19a shows the foam half-shell preform before cutting, while Figure 19b shows the desired final sections for robots R1 and R2. Multiple trials included adjustments to cutting speed as well as changes to the tooling design and robot offset. These adjustments did not produce clean cuts in the robotic cell. Figure 20 highlights the effects of cutting forces on 3D-shaped foam. Due to its softness and flexibility, the foam cannot withstand the localized pressure from the blade, which exceeds its minimal elastic deformation threshold, resulting in tearing rather than clean separation.

Several potential causes of the cutting failure were investigated. The oscillating blade was replaced to exclude blade wear as the primary cause, and the cutting tool was redesigned to improve guidance and support of the foam during the operation. Adjustments to robot speed and tool offset were also evaluated. Despite these modifications, sufficiently clean and repeatable cuts could not be achieved using the oscillating-blade configuration.

An alternative cutting method based on a heated Nichrome wire was therefore investigated. Figure 21 presents the corresponding experimental setup. Joule heating of the wire enables the foam to be separated with substantially lower mechanical cutting forces, thereby reducing deformation of the compliant workpiece.

Preliminary manual experiments produced cleaner and more consistent cuts than those obtained with the oscillating blade. The proposed setup includes a thermocouple for temperature monitoring, while the main controllable process parameters include electrical current, applied voltage, wire tension, and robot travel speed. Maintaining sufficient and stable wire tension is particularly important to limit wire deformation and reduce the risk of breakage during cutting.

Different Nichrome wire diameters were evaluated, with a 36-gauge wire (0.19 mm diameter) providing the most promising results among the tested configurations. Further investigation is required to determine the appropriate compromise between wire mechanical strength, thermal response, and cutting resistance. The effect of the resulting surface quality on subsequent assembly and welding performance also remains to be quantified. Future work will therefore focus on characterizing the effects of wire diameter, electrical current, and robot travel speed on cutting quality and on the mechanical performance of the resulting welded joints.

## 5. Conclusions

Aerospace manufacturing is characterized by low production volumes, substantial geometric variability, and stringent process requirements, making conventional robotic automation difficult to implement economically. In this context, the manufacturing of thermoplastic composite fluid-transfer ducts remains largely dependent on manual operations. This work investigated the feasibility of increasing the level of automation through the development of a dual-arm robotic manufacturing cell, with emphasis on process characterization, preliminary mechanical and functional assessment, and the initial development of a numerical pipeline for automated trajectory generation.

Experimental characterization of the laser transmission welding process showed that, among the evaluated parameter combinations, a robot travel speed of 12 mm/s and a laser power of 100 W produced the highest average breaking load, approximately 1.5 kN. These conditions were subsequently used as nominal parameters for manufacturing three-dimensional duct geometries. Reinforced thermoplastic ducts were successfully produced in automatic operation, providing a limited proof of concept for applying the robotic approach to both straight and curved components. Preliminary sealing experiments further showed that the manufactured ducts could withstand internal pressures of up to 15 psi without significant structural degradation, although localized leakage was observed, and additional quantitative characterization remains necessary.

Alternative strategies for cutting compliant foam components were also investigated. Cutting with an oscillating blade resulted in substantial deformation and tearing of the material, even after modifications to the cutting parameters and tooling. A heated Nichrome wire was therefore evaluated as an alternative. Preliminary experiments using a 36-gauge wire with a diameter of 0.19 mm produced cleaner and more consistent cuts, indicating that this approach is a promising candidate for future integration into the robotic manufacturing cell.

In parallel with the experimental development of the cell, an initial numerical pipeline was developed to automatically generate manufacturing trajectories from CAD geometry. The proposed approach extracts geometric information from STEP models, constructs local trajectory frames, and generates discrete robot poses that can subsequently be transferred to an offline programming environment for simulation and robot-code generation. Future work will focus on completing and validating this automated trajectory-generation framework for more complex duct geometries, further integrating it with the dual-arm robotic cell and quantitatively characterizing the welding, sealing, and foam-cutting processes. These developments are intended to further reduce manual programming and process adaptation requirements toward flexible, automated manufacturing of thermoplastic composite ducts for aerospace applications.

## Figures and Tables

**Figure 1:**
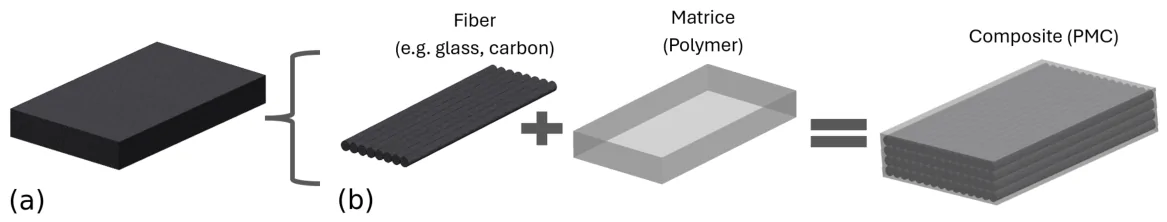
(**a**) Example of a raw composite plate and (**b**) distinction between the constituent elements of a composite material, inspired from [10].

**Figure 2:**
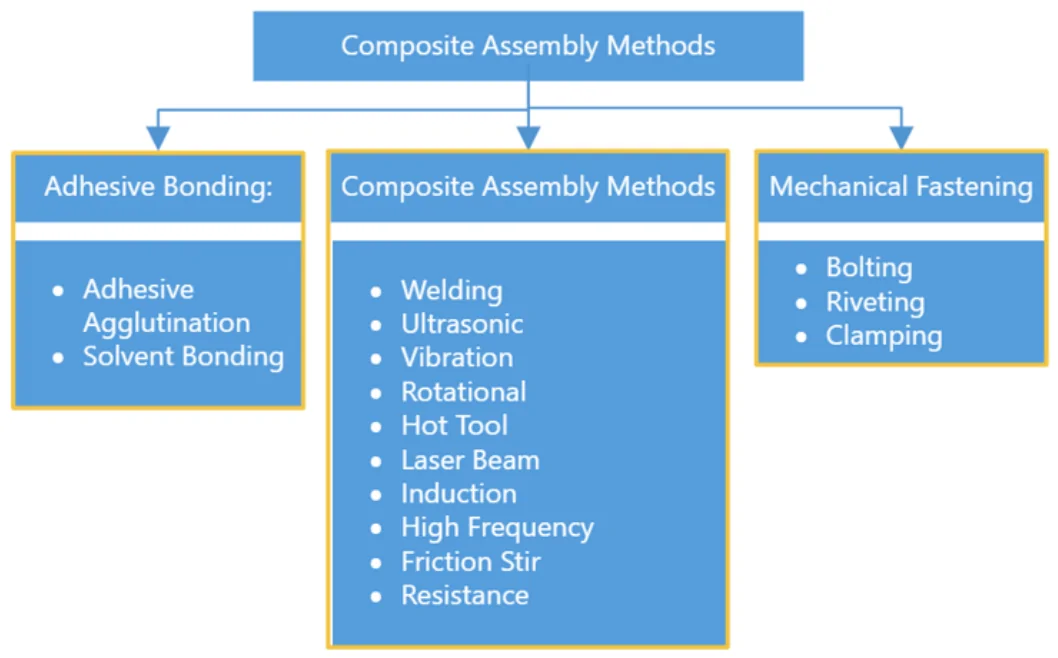
Joining methods for thermoplastic materials, adapted from [11].

**Figure 3:**
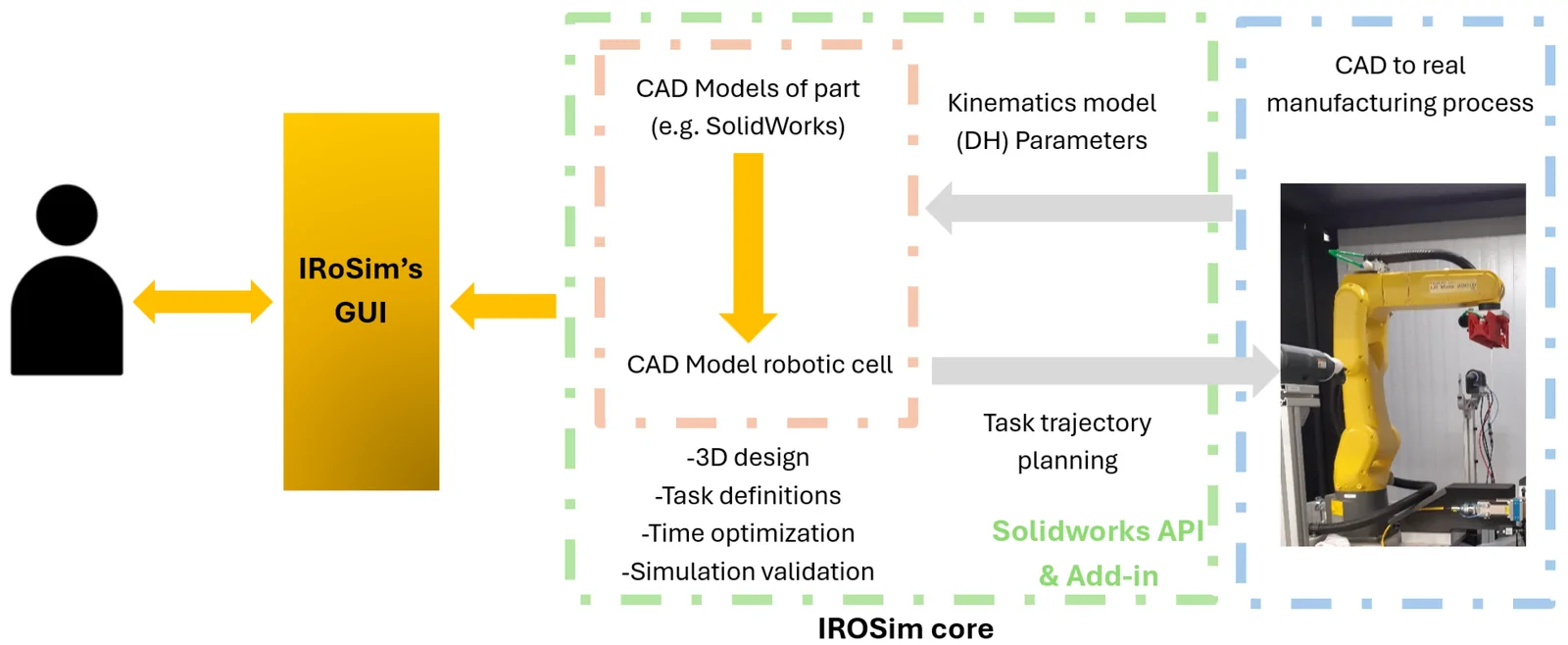
IRoSim offline programming pipeline using SolidWorks, inspired from [15]. The colored dashed frames distinguish the CAD model, IRoSim core and physical manufacturing stages, while the arrows indicate the direction of information flow.

**Figure 4:**
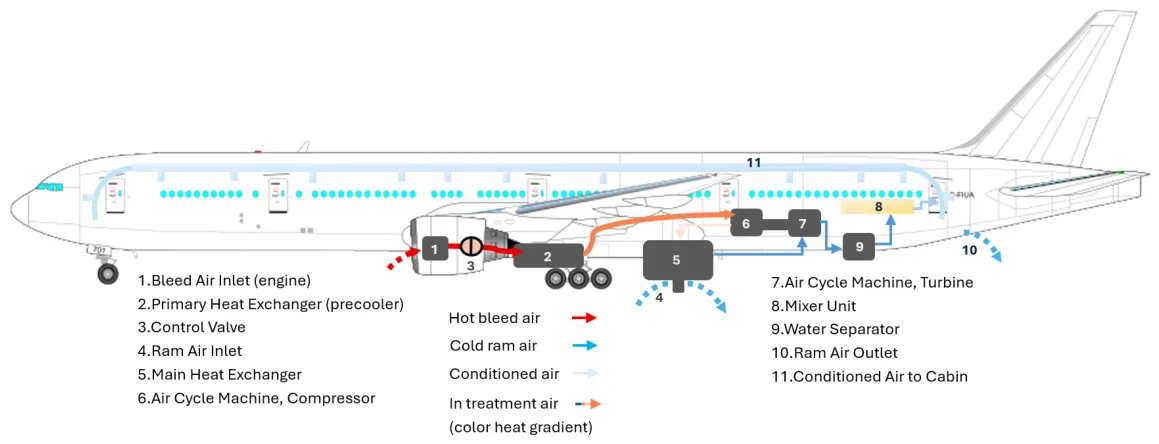
Fluid-transfer duct system in an aircraft, inspired from [26].

**Figure 5:**
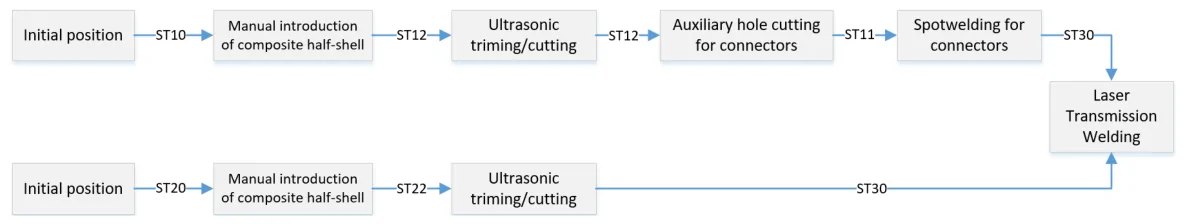
Manufacturing sequence for the production of a composite duct sample with an auxiliary connector.

**Figure 6:**
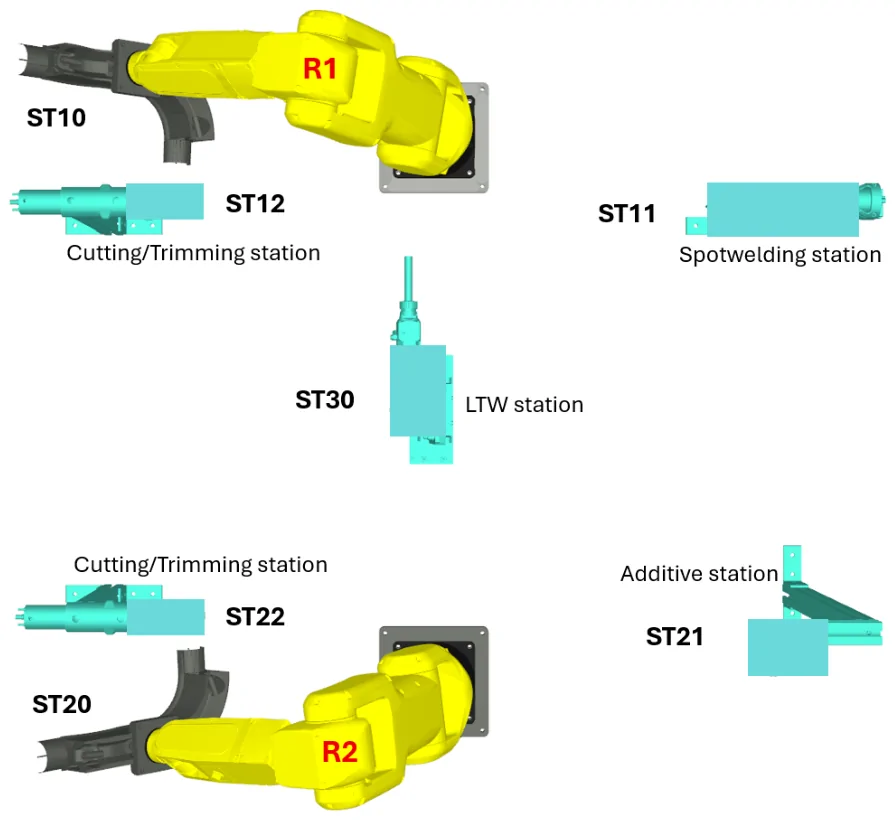
Elevation view of the cell layout showing the operating stations and their respective configurations.

**Figure 7:**
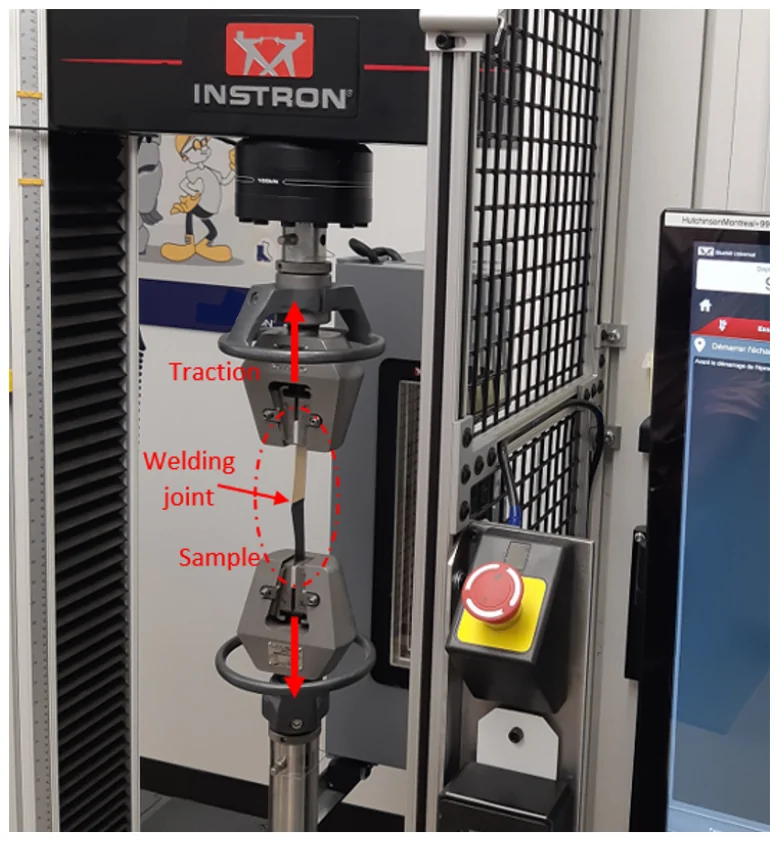
Test bench used for tensile testing.

**Figure 8:**
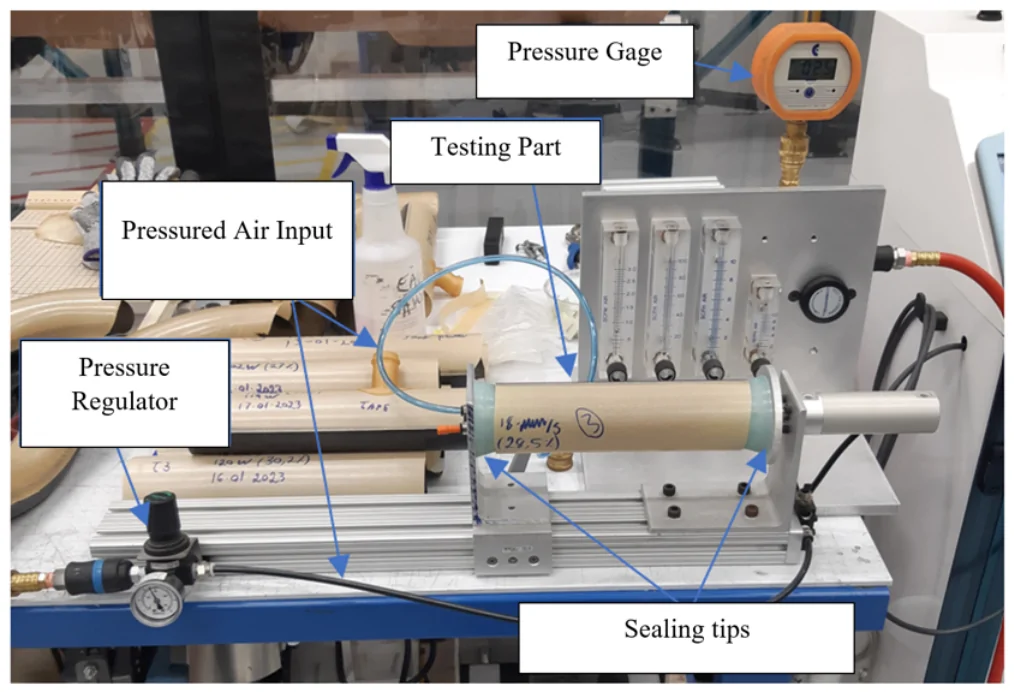
Test bench used for duct leakage testing.

**Figure 9:**
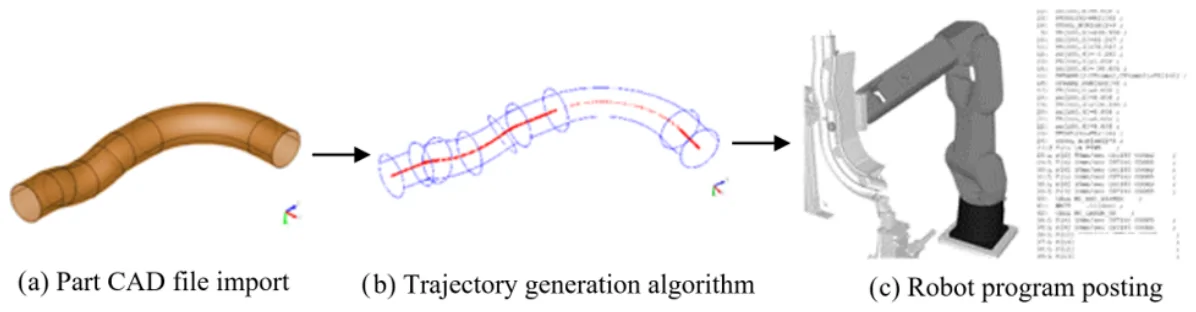
Main steps of the numerical tool for automated path generation.

**Figure 10:**
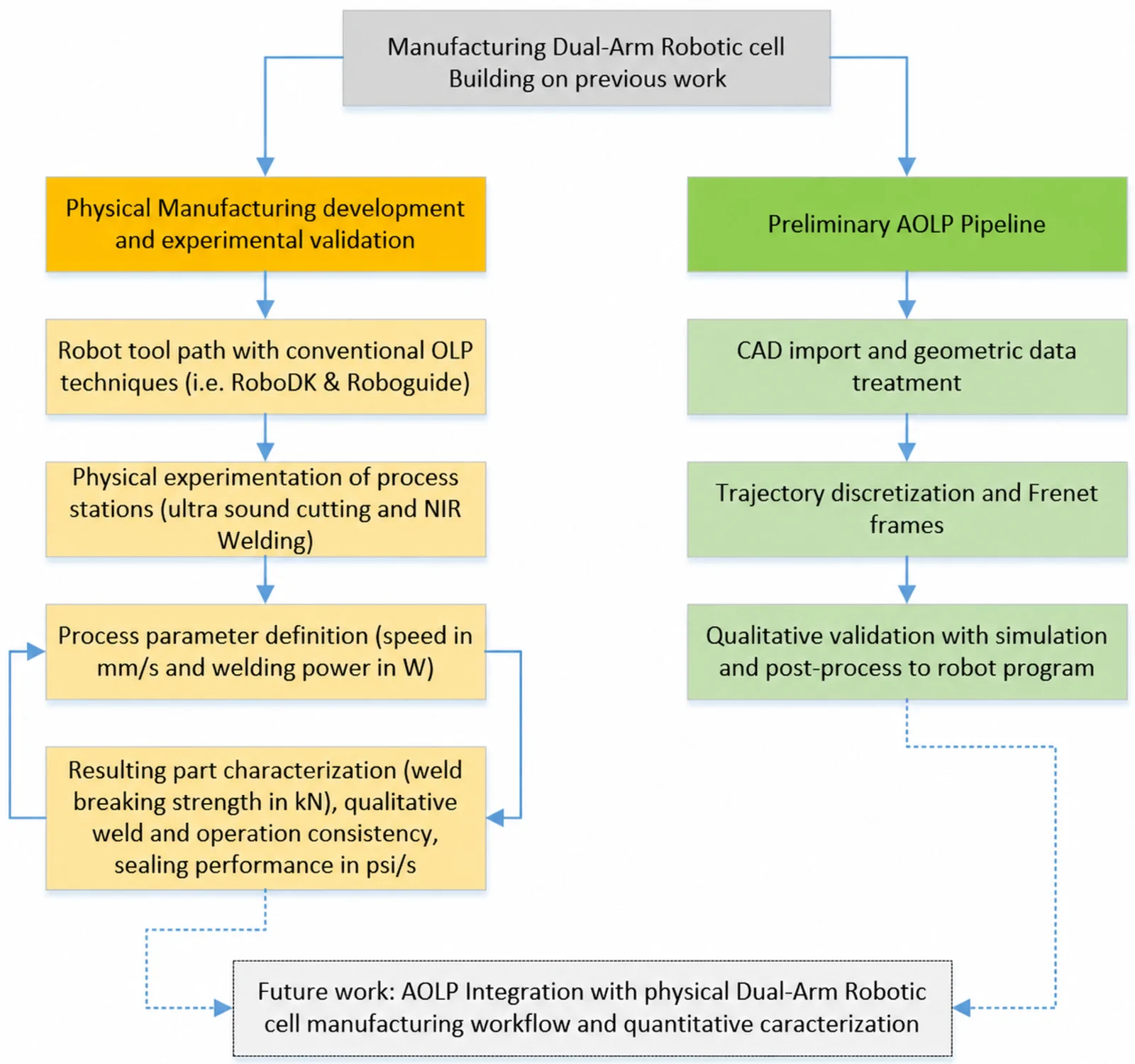
Technical roadmap of this study, building on the developments reported in Refs. [7,8]: physical process development and experimental validation (**left**) and preliminary AOLP trajectory generation and simulation (**right**).

**Figure 11:**
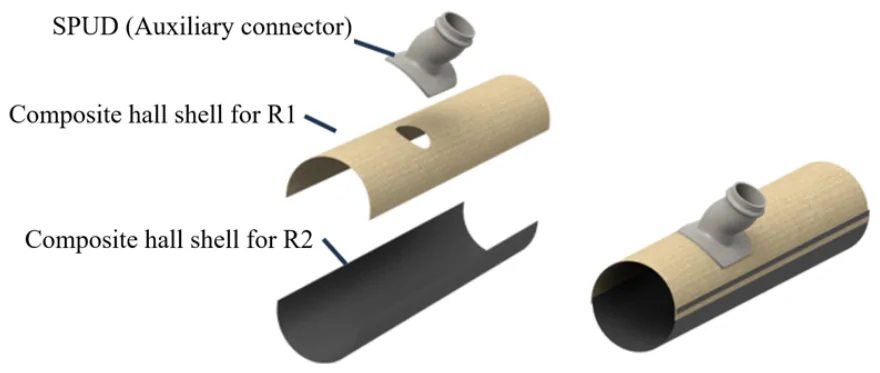
Three-dimensional model of a reinforced thermoplastic duct: exploded view (**left**) and assembled configuration (**right**).

**Figure 12:**
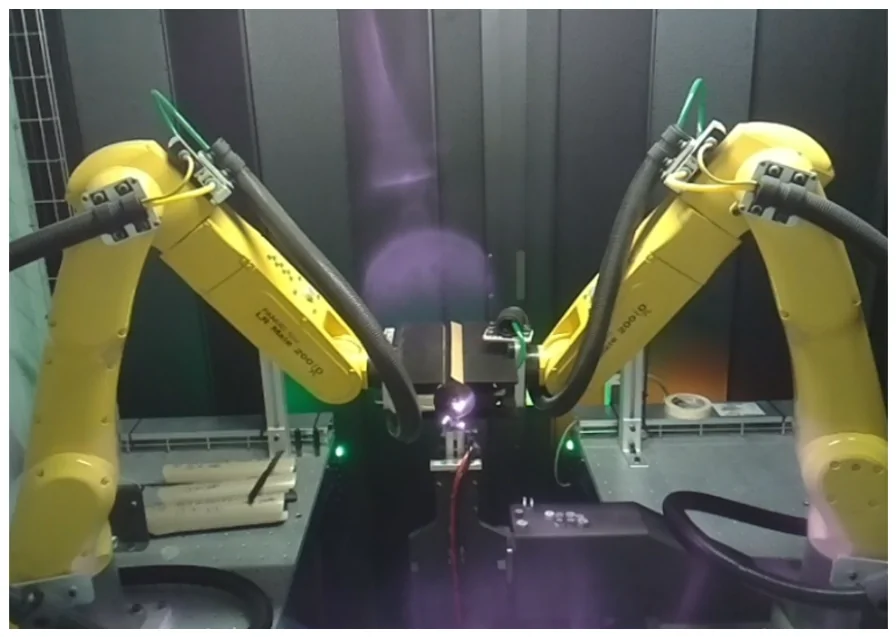
Robots R2 (**left**) and R1 (**right**) during positioning and initiation of the laser-welding process.

**Figure 13:**
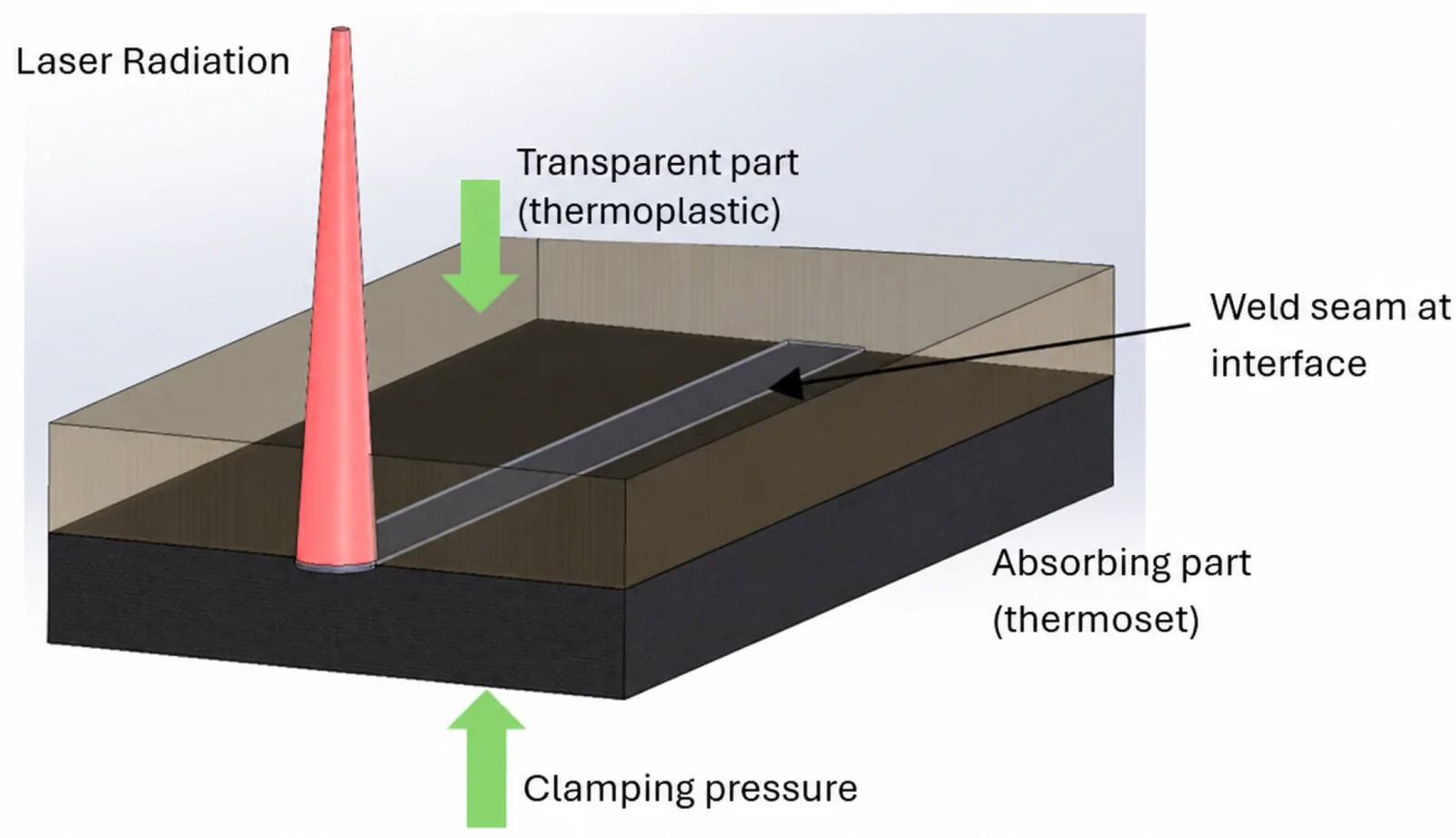
Laser transmission welding process for thermoplastic composite parts, inspired from [28].

**Figure 14:**
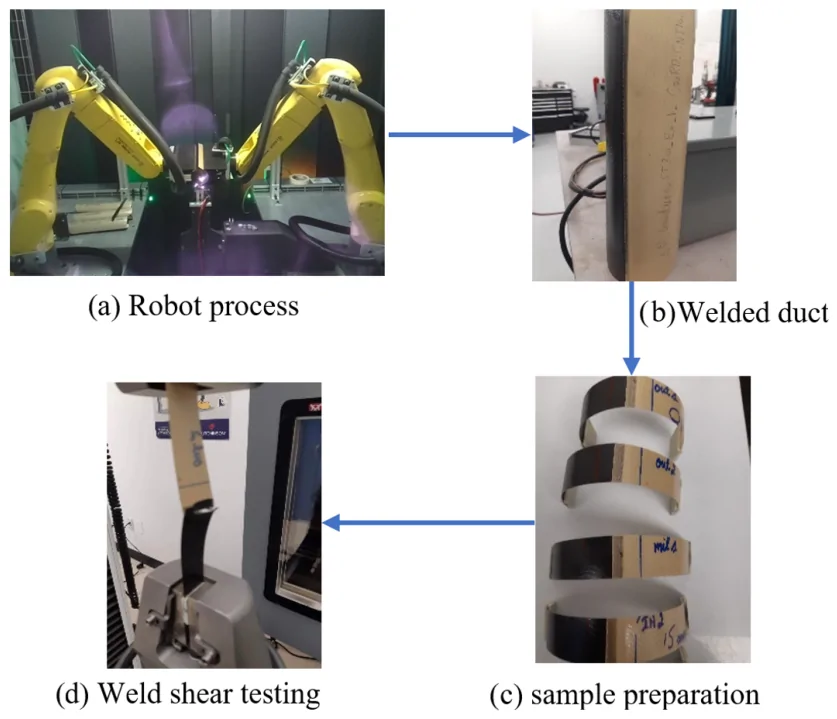
Workflow used for manufacturing and mechanical testing of welded samples.

**Figure 15:**
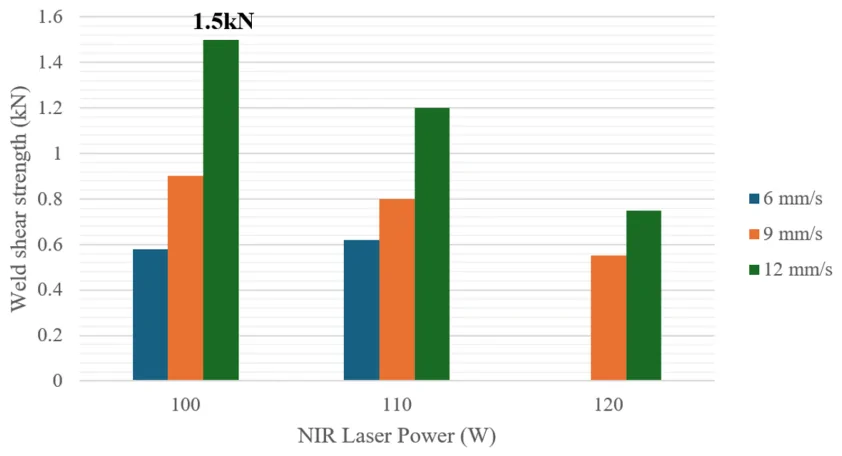
Mean joint-breaking load (kN) for the tested laser-power and robot-travel-speed combinations (four specimens per combination).

**Figure 16:**
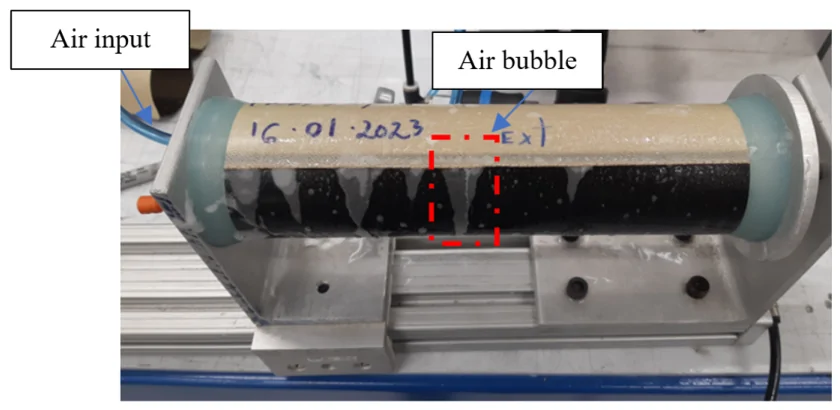
Localized leakage detected by soap-bubble inspection at 7.5 psi.

**Figure 17:**
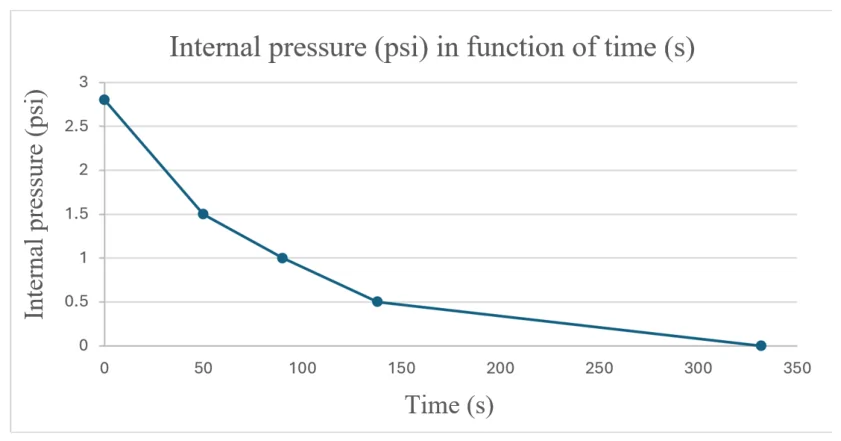
Measured internal pressure decay over time.

**Figure 18:**
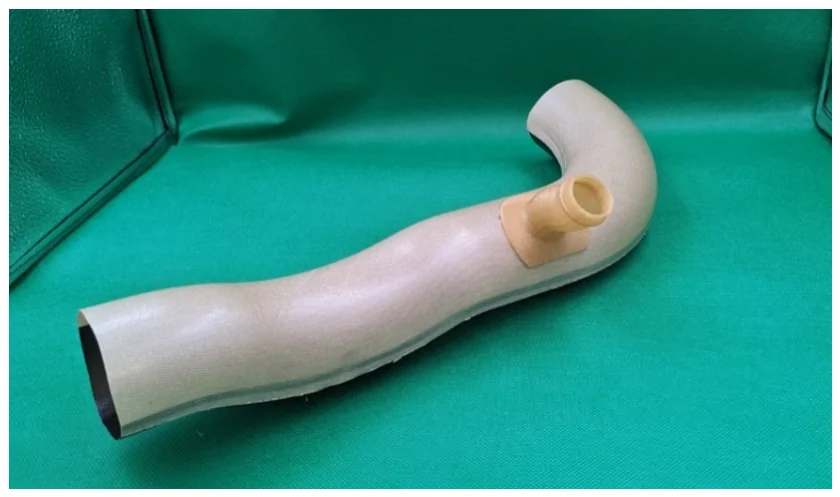
Resulting reinforced thermoplastic 3D curved test part.

**Figure 19:**
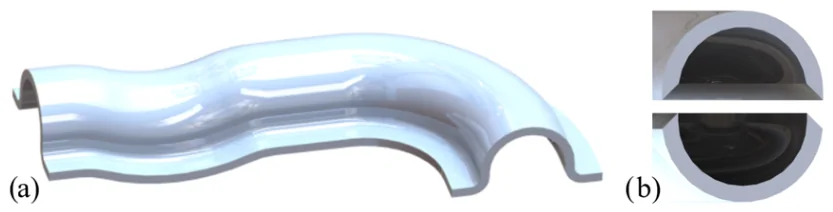
(**a**) Foam half-shell preform and (**b**) final cutting result.

**Figure 20:**
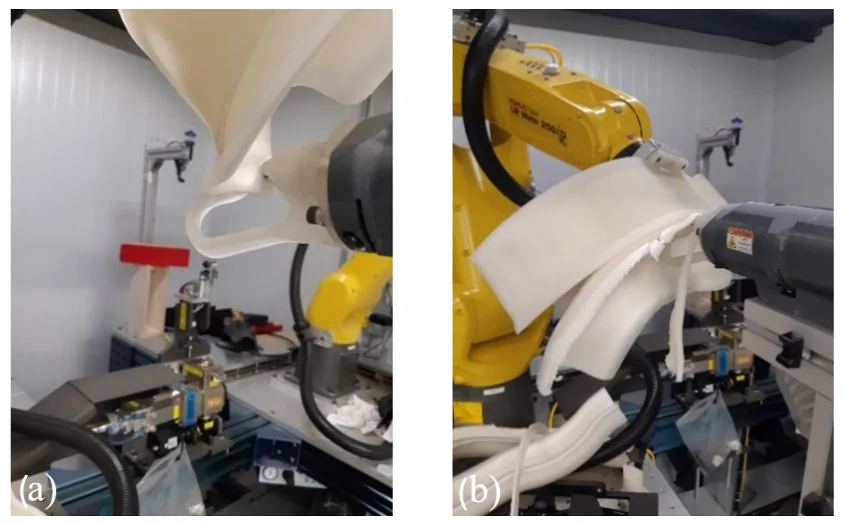
Failures during cutting of 3D-shaped foam: (**a**) initial snagging and deformation of the half-shell; (**b**) progression of tearing in the half-shell.

**Figure 21:**
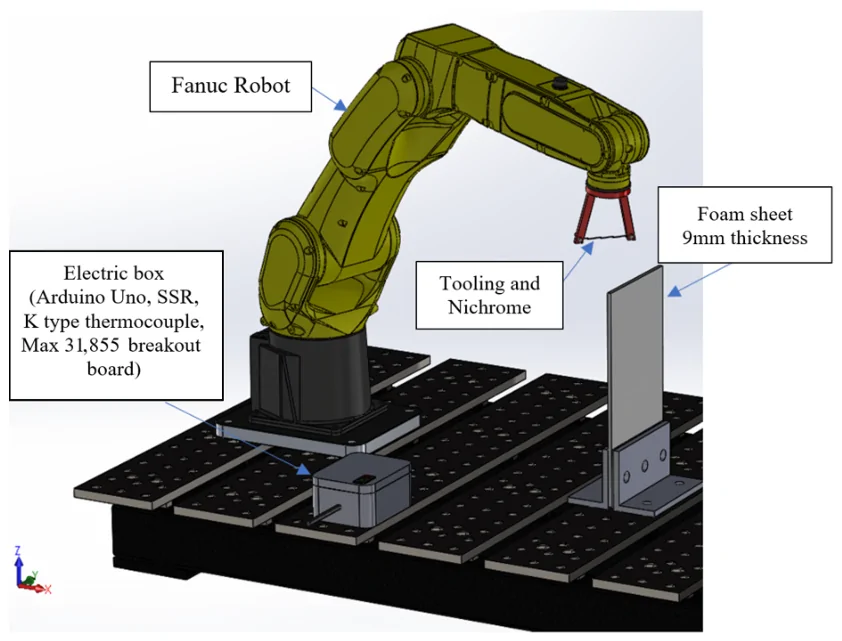
Test setup for foam cutting using a Nichrome wire.

**Table 1:** Synthesis of representative approaches to robotic offline programming, performance analysis, and manufacturing-system optimization.

Study	Programming Input or Method	Performance or Optimization Focus	Principal Limitation Relative to the Present Application
Wu et al. [13]	SolidWorks-based OLP for robotic welding	Virtual programming and generation of executable robot programs	Reliance on proprietary CAD software and user interaction
Toledano-García et al. [14]	Two- and three-dimensional interpolation of meshed CAD geometry	Automated generation of waypoints over planar and spatial surfaces	Dependence on commercial CAD software and MATLAB; no complete cell-level workflow
Baizid et al. [15]	SolidWorks API and IRoSim platform	Integrated cell design, path planning, simulation, and optimization	Proprietary-software dependence and trajectory redefinition when the part geometry changes
Rodrigues et al. [16]	OLP combined with structured-light three-dimensional vision	CAD-to-physical-cell registration and trajectory adaptation from limited user-defined waypoints	Application-specific implementation; no autonomous extraction of complex duct trajectories
Bedaka and Lin [19]	Programmatic processing of CAD geometry and robot simulation	Autonomous path generation without direct manipulation of STEP data	Limited reporting of physical-process and cell-level performance metrics
Chen et al., Lin and Lee and Mineo et al. [20,21,22]	CAD-driven path generation for painting, machining, and non-destructive testing	Application-specific coverage, path adaptation, or inspection planning	Geometric and process constraints remain task specific
Babcinschi et al. [23]	CAD/CAM positions combined with demonstrated orientations and velocities; PathML interoperability	Position and orientation error, simulation checks, and physical validation in welding and adhesive deposition	Requires an expert demonstration, tracking hardware, and virtual-to-real calibration
Zhang et al. [25]	Sequential optimization and time-dependent reliability assessment	Computational efficiency and reliability under dynamic uncertainty	General design-optimization method rather than a robot-programming framework; requires uncertainty and performance models
Kawabe et al. [24]	RRT* motion planning, NSGA-II, and surrogate-assisted cell-layout optimization	Cell area, robot operation time, computation time, and surrogate-model accuracy	Does not address manufacturing-process quality or coordinated dual-arm process execution
Present work	STEP/OCCT-based construction of discrete position and orientation frames, followed by RoboDK transfer	Preliminary path completeness and coherence; separate experimental evaluation of weld strength and sealing performance	Quantitative trajectory accuracy, programming-time reduction, optimization, and full integration with the physical cell remain future work

## Data Availability

The data presented in this study are available from the corresponding author upon reasonable request and subject to the confidentiality restrictions imposed by the non-disclosure agreement (NDA) with the industrial partner. The data are not publicly available due to these confidentiality restrictions.
